# Effects of neuromuscular electrical stimulation on gait performance in chronic stroke with inadequate ankle control - A randomized controlled trial

**DOI:** 10.1371/journal.pone.0208609

**Published:** 2018-12-10

**Authors:** Yea-Ru Yang, Pei-Ling Mi, Shih-Fong Huang, Shiu-Ling Chiu, Yan-Ci Liu, Ray-Yau Wang

**Affiliations:** 1 Department of Physical Therapy and Assistive Technology, National Yang-Ming University, Taipei, Taiwan; 2 Department of Neurosurgery, Neurological Institute, Taipei Veterans General Hospital, Taipei, Taiwan; Bern University of Applied Science, SWITZERLAND

## Abstract

Neuromuscular electrical stimulation (NMES) has been used to improve muscle strength and decrease spasticity of the ankle joint in stroke patients. However, it is unclear how NMES could influence dynamic spasticity of ankle plantarflexors and gait asymmetry during walking. The study aimed to evaluate the effects of applying NMES over ankle dorsiflexors or plantarflexors on ankle control during walking and gait performance in chronic stroke patients. Twenty-five stroke participants with inadequate ankle control were recruited and randomly assigned to an experimental or a control group. The experimental group received 20 minutes of NMES on either the tibialis anterior muscle (NMES-TA) or the medial gastrocnemius muscle (NMES-MG). The control group received 20 minutes of range of motion and stretching exercises. After the 20 minutes of NMES or exercises, all participants received ambulation training for 15 minutes. Training sessions occurred 3 times per week for 7 weeks. The pre- and post-training assessments included spatio-temporal parameters, ankle range of motion, and dynamic spasticity of ankle plantarflexors during walking. Muscle strength of ankle dorsiflexors and plantarflexors as well as static spasticity of ankle plantarflexors were also examined. The results showed that the static and dynamic spasticity of ankle plantarflexors of the NMES-TA group were significantly decreased after training. Reduction in dynamic spasticity of ankle plantarflexors of the NMES-TA group was significantly greater than that of the NMES-MG group. When compared to the control group, the NMES-TA group had greater improvements in spatial asymmetry, ankle plantarflexion during push off, and muscle strength of ankle dorsiflexors, and the NMES-MG group showed a significant decrease in temporal asymmetry. In summary, NMES on ankle dorsiflexors could be an effective management to enhance gait performance and ankle control during walking in chronic stroke patients. NMES on ankle plantarflexors may improve gait symmetry.

## Introduction

Inadequate ankle control during walking has been identified as one key factor contributing to gait dysfunction, such as decreased gait speed and symmetry after stroke [1,2]. Many stroke rehabilitation programs have thus targeted on the improvement of ankle control.

Weakness of ankle dorsiflexors with or without plantarflexors spasticity could result in insufficient ankle dorsiflexion at heel strike and in swing phase during walking [3], and muscle weakness and spasticity of ankle plantarflexors could lead to insufficient ankle plantarflexion in terminal stance or push off [4,5]. Studies have shown that spasticity of ankle plantarflexors associated with the decreased gait velocity and increased gait asymmetry in stroke patients [6]. Dynamic spasticity of ankle plantarflexors, which is locomotor-specific [7], was positively correlated with gait velocity and could explain 53% of the variance in spatial gait asymmetry [3]. Lin et al. also indicated that isometric muscle strength of ankle dorsiflexors was the primary determinant of gait velocity and temporal gait asymmetry [3]. On the other hand, Kim and Eng identified the isokinetic torque of ankle plantarflexors related to gait velocity and explained 72% of its variance [8]. Therefore, increasing ankle muscle strength and decreasing plantarflexors spasticity are important for improving gait velocity and symmetry in people with stroke.

Traditional strategies for ankle control include muscle performance training, flexibility exercise, spasticity management, and modalities such as electrical stimulation (ES) and electromyographic (EMG) biofeedback devices [9]. Neuromuscular electrical stimulation (NMES), which provides ES on nerve fibers in healthy or denervated muscles to induce muscle contractions, is the most widely used modality in clinics and has been adopted to manage the drop foot for stroke patients since early 1960s [10]. The high peak current with specific waveform of NMES can maximize the numbers of responding motor units and their firing rate leading to tetantic contractions and great forces [11]. Applying NMES may not only improve muscle strength but decrease spasticity of agonist or antagonist muscles possibly through maximal contraction inducing relaxation or reciprocal inhibition respectively [11–14]. Bakhtiary and Fatemy found that combining NMES on tibialis anterior with Bobath therapy significantly improved more in muscle strength of ankle dorsiflexors and static spasticity of ankle plantarflexors than Bobath therapy alone [13]. Barth et al. also suggested that a 4-week daily EMG-triggered NMES intervention on tibialis anterior could improve active range of motion (AROM) of ankle dorsiflexion and plantarflexion, balance and gait performance in chronic stroke patients [15]. However, the effects of NMES to plantarflexors on gait performance are not immediately known although the possible reciprocal inhibition neural mechanism has been suggested.

Although the outcome of using NMES in stroke rehabilitation is promising, studies that examined the effect of NMES on dynamic spasticity during walking and gait asymmetry in stroke patients are limited. Since spasticity is velocity-dependent, measuring static spasticity following NMES may not explain the effect of NMES on dynamic spasticity during walking. The purpose of this study was to investigate the effects of applying NMES over ankle dorsiflexors or plantarflexors on ankle control and gait performance in chronic stroke patients. This information can provide the possible mechanisms of NMES involved in ankle control and enhance the development of training strategy in stroke rehabilitation.

## Methods

### Participants

**Fig 1:**
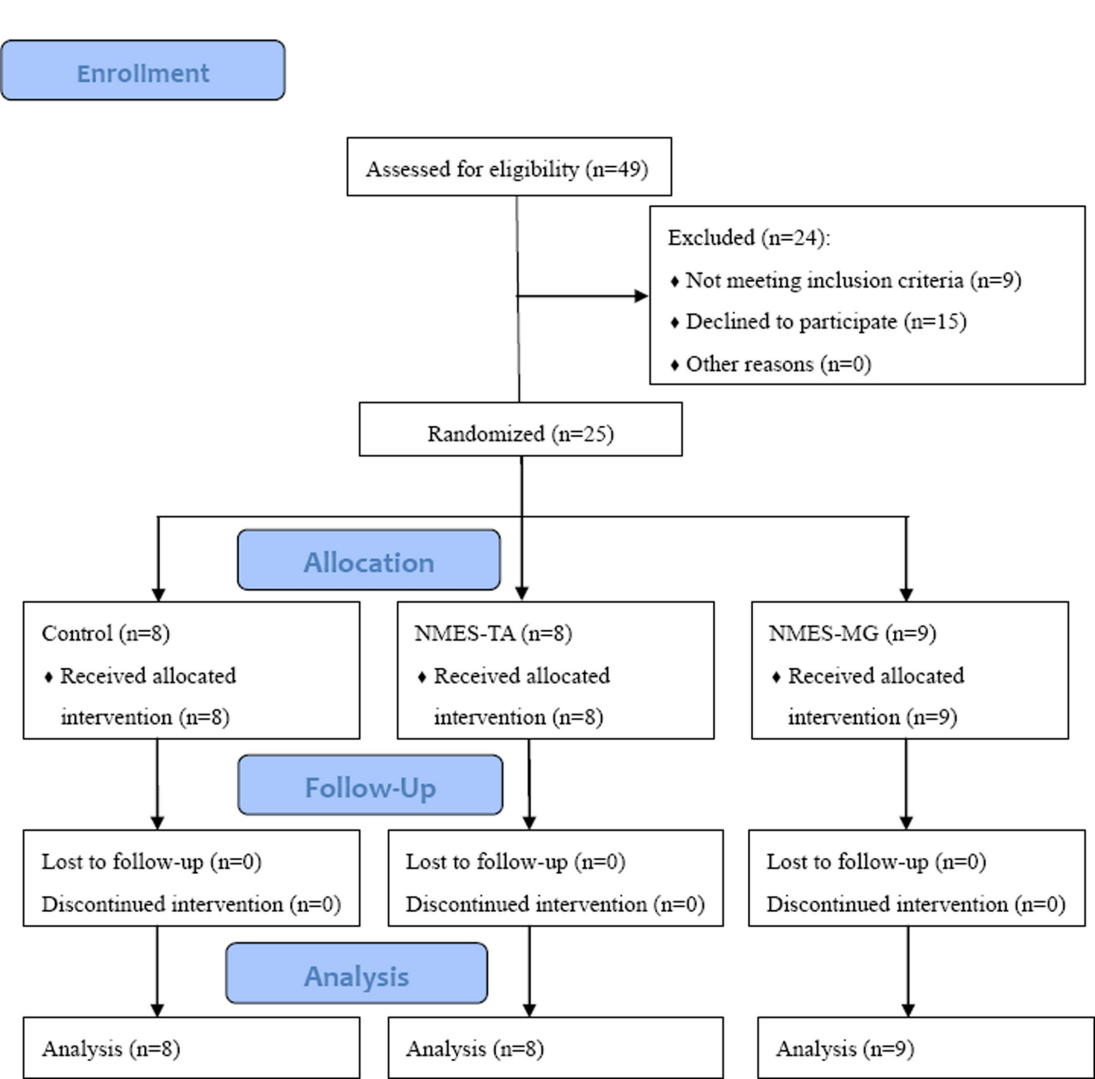
Study flowchart.

Participants who had suffered a stroke were recruited from Taipei Veterans General Hospital, Taipei Taiwan (R.O.C) from August 2013 to June 2014. The diagnosis, age, sex, stroke type, lesion side, and post onset duration of stroke were obtained from patient interviews and medical charts. To be included in the study, participants with stroke had to satisfy the following criteria: (1) diagnosis of first-ever stroke with unilateral motor deficits at least 6 months, (2) with inadequate ankle control during gait (defined as maximum position of ankle dorsiflexion less than -5° at heel strike and plantarflexion less than 10° at push off, 0° was set as neutral position), (3) with passive range of motion (PROM) of ankle dorsiflexion at least to neutral position (defined as 0°), (4) ability to walk at least 10 m with or without assistive devices, and (5) a detectable surface EMG signal (>5 μV) from the tibialis anterior (TA) and medial gastrocnemius (MG) muscles of the affected leg. The exclusion criteria included (1) surface sensory loss of affected lower leg, (2) insufficient cognition to communicate (Mini-Mental State Examination < 24), (3) contraindications to NMES, such as a pacemaker or tumor, and (4) a history of orthopedic or other neurologic disorders affecting walking function (5) a history of surgery to correct drop foot. This study protocol was approved by the Institutional Review Board (IRB) of Taipei Veterans General Hospital on July 19^th^, 2013. The complete date range for patient recruitment and follow-up were between August 22^th^, 2013 and June 25^th^, 2014. All participants signed informed consent in advance. The registration of the trial was not done right after the IRB approval because the authors were less aware of the required prospective registration. However, this trial was registered in http://www.anzctr.org.au/ (ACTRN12617000786392) on May 29^th^, 2017. The authors confirmed that all ongoing and related trials for this intervention are registered as well as the CONSORT flowchart (Fig 1) and checklist.

### Experimental design

This was a single-blinded, parallel randomized, controlled trial study. Participants were randomized to one of three groups using block randomization with a block size of three: the NMES-TA group (NMES applied on tibialis anterior muscle), the NMES-MG group (NMES applied on medial gastrocnemius muscle), and the control group. Participants in the NMES groups received 20 minutes of NMES on either TA (NMES-TA) or MG (NMES-MG) and then 15 minutes of ambulation training. Participants in the control group received 20 minutes of range of motion and stretching exercises, followed by 15 minutes of ambulation training. Ambulation training focused on ankle movement and ankle control with verbal cues. All training sessions occurred 3 times per week for 7 weeks which were conducted by the same physical therapist. The pre- and post-training assessments completed within 7 days before and after the 21 training sessions. All training sessions and assessments were one-on-one basis which took place in a quiet, comfortable, and private room. Assessment data were obtained by another physical therapist who was blinded to the group assignment.

### NMES training protocol

An EMG-triggered NMES (Myomed 932, Enraf Nonius, Netherlands) with two surface electrodes was used for ES in this study. Participants were in sitting position with feet off the ground during NMES sessions. For the NMES-TA group, the cathode electrode was placed on the motor points of TA, and the anode electrode was located at the mid-muscle belly on one third of the line between fibular head and medial malleolus. For the NMES-MG group, both electrodes were placed on the muscle belly of MG. The cathode electrode was located at about 2 cm medial to the midline of shank and 5 finger-widths distal to the popliteal fossa, and the anode electrode was placed on 2 cm distal to the cathode electrode. The reference electrode was placed on the distal part of the targeted muscles. The frequency of NMES was set at 50 Hz with a 0.2 ms pulse width. Biphasic square wave was chosen to provide a specific waveform, and the stimulation duty cycle was 5:15 (on:off) in seconds for 20 minutes. The intensity of stimulation was set from 50 mV to 0 mV to induce full range of motion of ankle dorsiflexion or plantarflexion without causing any discomfort [11].

EMG signals of the targeted muscles were recorded and displayed on the screen of NMES machine with auditory feedbacks. EMG signals of maximal voluntary contraction of ankle dorsiflexion (NMES-TA group) or plantarflexion (NMES-MG group) subtracting 2 uV was used as the initial training goal in every session. When receiving NMES, participants were asked to actively dorsiflex (NMES-TA group) or plantarflex (NMES-MG group) the ankle joint to reach the initial training goal that activated the ES. After completing five successful cycles of active ankle dorsiflexion or plantarflexion, the training goal was increased by 2 uV progressively. The NMES training lasted for 20 minutes, followed by ambulation training focusing on ankle control for another 15 minutes. Verbal cues were provided to enhance ankle movement during walking. For instance, participants were instructed to “elevate your foot more (dorsiflexion)”, “please do more foot eversion”, and “heel contacts floor first instead of forefoot”.

In the control group, participants received exercise of the affected lower extremity for 20 minutes, including stretching for 5 minutes, PROM exercise for 5 minutes, and AROM exercise for 10 minutes. Following the exercise, a 15 minutes of ambulation training, which was the same as described in the NMES groups, was performed.

## Outcome measurements

### Primary outcomes

Gait performance as the primary outcome was measured by using the GAITRite system (CIR system, Inc., Havertown, Pennsylvania) which is a 4.57 m long and 0.9 m wide walkway containing pressure-sensitive sensors with an active area of 3.66 m long and 0.61 m wide. Participants were instructed to walk along the walkway at their comfortable speed, and a walking assistive device was allowed if necessary, except an ankle-foot orthosis. Gait velocity, cadence, and step length of the affected and unaffected limbs were calculated from the contact time and location of each footfall in the system. In addition, the spatial and temporal asymmetry ratios were calculated using the following formulas [3]. A high value of the ratio indicates a greater degree of asymmetry.

$$\text{Spatial asymmetry ratio}=\left|1-\frac{\text{step length}\;(\text{affected})}{\text{step length}\;(\text{unaffected})}\right|$$

$$\text{Temporal asymmetry ratio}=\left|1-\frac{\text{single support time}\;(\text{affected})}{\text{single support time}\;(\text{unaffected})}\right|$$

### Secondary outcomes

#### Static and dynamic spasticity of ankle plantarflexors during gait

The static spasticity of ankle plantarflexors was measured in the supine position using Modified Ashworth Scale (MAS) [16]. The spasticity index, which is the slope of a linear relationship between EMG activities of a muscle and its lengthening velocities, was used to determine the dynamic spasticity of MG during stance phase of a gait cycle. A positive slope represents a velocity-dependent muscle activation, indicating hyperactive stretch reflexes or spasticity [7].

For measuring the dynamic spasticity, EMG activities of MG were measured concurrently when the participant walked along the GaitRite walkway. To obtain the lengthening velocity of MG, the displacements of the knee and ankle joints were measured and used to configure the model developed by Winter and Scott [17]. Two twin-axis electronic goniometers (SG110, Biometrics Ltd, UK) (SG110, Biometrics Ltd, UK) were placed on the ankle and knee joints to measure the displacements. Two footswitches were placed under the toe and heel to determine the stance and swing phases of a gait cycle. Finally, EMG activities and lengthening velocities of MG during stance phase was plotted as linear relationship to define its spasticity index as suggested by Lamontagne et al. [3,7].

#### Maximum position of the ankle joint during gait

The Maximal position (MP) of dorsiflexion at heel strike and plantarflexion at push off were measured by the electronic goniometer placed on the ankle joint during walking as described above. At heel strike, a positive value of MP indicates ankle dorsiflexion, and a negative value indicates ankle plantarflexion. The MP of the ankle joint in push off was defined by changes of ankle joint position from heel off to toe off. Therefore, a positive value of MP during push off indicates ankle plantarflexion, and a negative value indicates ankle dorsiflexion. The coefficients of variance (CV) of MP at heel strike and in push off were also evaluated to further identify the ability of ankle control [4].

#### Muscle strength

Maximal isometric muscle strength of ankle dorsiflexors and plantarflexors in affected leg were measured using a handheld dynamometer (Power Track II; Jtech Medical Industries Inc., Herber City, UT). The ankle dorsiflexors were examined in the supine position with the hip and knee flexed to 90° and supported by a wooden block [18]. The ankle plantarflexors were evaluated in the supine position with both hip and knee extended [19, 20]. The examiner held the handheld dynamometer stationary while the participant exerted a maximal force against it for 5 seconds.

### Statistical analysis

A Wilcoxon signed-rank test was used to detect the pre- and post-training differences for within-group comparisons. To evaluate the training effects for between-group comparisons, a percentage of change score was calculated by subtracting the pre-training data (baseline) from the post-training data and divided by the pre-training data. The demographic, baseline, and percentage of change score were compared among three groups using a Kruskal-Wallis test. Follow-up pairwise comparison was conducted using a Dunn-Bonferroni post-hoc method. All statistical analyses were performed with the SPSS 20.0 software (SPSS Inc., USA).

## Results

There were 25 subjects participated and completed the study protocol, 8 participants in the NMES-TA group, 9 participants in the NMES-MG group, and 8 in the control group. For the demographic and baseline data, no significant group differences were detected (Tables 1 and 2). After 7 weeks of training, the gait velocity and cadence of all three groups increased, however, significant post-training improvements and group differences in change scores were not detected (Table 3). For the step length after training, the NMES-TA group demonstrated a significant increase in the affected limb (p = 0.036), and the NMES-MG group showed significant increases in both the affected limb (p = 0.011) and unaffected limb (p = 0.028). The pre- and post-training differences in the control group were not significant. There were no significant group differences in change scores regarding the step length. The spatial asymmetry ratio of all three groups were not significantly reduced after training, however, change scores of it was significantly decreased in the NMES-TA group as compared to the control group (p = 0.018). For the temporal asymmetry ratio, the NMES-MG group demonstrated a significant decrease after training (p = 0.011) which was not found in the control group and NMES-TA groups. The decrease in temporal asymmetry ratio in the NMES-MG group was significantly more than that of the control group (p = 0.019).

**Table 1:** Demographic characteristics of the participants.

	**Control (N = 8)**	**NMES-TA (N = 8)**	**NMES-MG (N = 9)**	**p value**
**Age (years)**	50.8 ± 3.8	49.6 ± 3.6	56.1 ± 2.1	0.364
**Post-onset duration (mos)**	31.8 ± 6.1	48.0 ± 9.1	41.8 ± 6.4	0.380
**MMSE**	28.1 ± 0.5	28.4 ± 0.3	27.8 ± 0.8	0.948
**Gender**				0.262
Male	6 (75%)	6 (75%)	9 (100%)	
Female	2 (25%)	2 (25%)	0 (0%)	
**Type of stroke**				0.723
Infarction	4 (50%)	4 (50%)	6 (66.7%)	
Hemorrhage	4 (50%)	4 (50%)	3 (33.3%)	
**Side of stroke**				0.965
Right	3 (42.9%)	4 (50%)	5 (50%)	
Left	5 (57.1%)	4 (50%)	4 (50%)	
**Assistive device**				
Cane	3	1	2	0.498
Quadricane	2	1	1	0.699
AFO	2	2	0	0.262

Abbreviations: mos: months; MMSE: mini-mental state examination; AFO: ankle-foot orthosis. p value, intergroup difference

**Table 2:** Baseline data of the participants.

	Control (N = 8)	NMES-TA (N = 8)	NMES-MG (N = 9)	p value
**Gait performance**				
** **Velocity (cm/s)	50.5±7.5	48.3±8.3	49.1±7.9	0.930
** **Cadence (steps/min)	77.8±5.7	69.1±6.0	70.3±7.1	0.611
** **Step length (affected, cm)	40.7±4.4	39.8±4.2	45.1±4.5	0.689
** **Step length (unaffected, cm)	34.3±2.9	40.1±4.4	35.9±2.9	0.717
**Muscle strength**				
** **Ankle dorsiflexors strength (N)	89.9±11.4	76.5±14.0	92.9±9.1	0.458
** **Ankle plantarflexors strength (N)	112.9±16.5	104.4±9.6	110.9±8.2	0.804
**MAS (0–5)**	1.9±0.4	2.4±0.3	2.1±0.3	0.464
**Spasticity index**	3.4±4.2	9.8±2.5	7.2±2.8	0.749
**Maximum position during gait**				
** **CV of ankle dorsiflexion at HS (%)	10.5±1.9	7.2±1.5	10.1±2.9	0.405
** **CV of ankle plantarflexion in push off (%)	34.4±15.8	19.9±4.9	42.9±12.9	0.188
** **MP of dorsiflexion at HS (degree)	-21.9± 2.3	-25.3±3.6	-21.6±2.9	0.717
** **MP of plantarflexion in push off (degree)	-6.2± 1.0	-10.3±1.7	-10.1±1.9	0.165

Abbreviations: MAS: modified Ashworth scale; CV: coefficient of variation; HS: heel strike; TA: tibialis anterior; MG: medial gastrocnemius; MP: Maximum position. p value, intergroup difference

**Table 3:** Gait performance before and after training in 3 groups.

	Control (N = 8)		NMES-TA (N = 8)		NMES-MG (N = 9)	
	Pre	Post	Pre	Post	Pre	Post
Velocity (cm/s)	50.5±7.5	54.1±10.3	48.3±8.3	61.7±11.5	49.1±7.9	52.8±6.7
** **Change values (%)^a^		2.4±16.7		30.6±37.6		13.6±24.9
Cadence (steps/min)	77.8±5.7	77.3±7.0	69.1±6.0	76.5±7.3	70.3±7.1	72.5±4.9
** **Change values (%)^a^		-1.4±11.1		10.9±13.8		7.1±19.5
Step length (affected, cm)	40.7±4.4	43.5±5.3	39.8±4.2	46.9±4.9*	45.1±4.5	48.1±4.5*
** **Change values (%)^a^		5.3±11.3		18.4±15.8		7.5±10.5
Step length (unaffected, cm)	34.3±2.9	35.2±4.5	40.1±4.4	44.9±4.5	35.9±2.9	39.5±2.7*
** **Change values (%)^a^		0.5±16.4		15.2±24.6		11.8±16.1
Spatial asymmetry	0.25±0.08	0.35±0.10	0.19±0.06	0.09±0.04	0.19±0.05	0.15±0.04
** **Change values (%)^a^		121.7±183.2		-41.9±64.0#		-18.8±45.5
Temporal asymmetry	0.27±0.05	0.37±0.04	0.23±0.04	0.25±0.06	0.32±0.04	0.23±0.04*
** **Change values (%)^a^		65.2±87.2		16.8±92.8		-25.6±22.9#

^a^ Change values were calculated by subtracting the pre-training data (baseline) from the post-training data divided by the pre-training data and expressed as %

*, p<0.05 for intra-group difference

#, p<0.05 compared with control group

The MAS and dynamic spasticity of ankle plantarflexors decreased in all three groups after training (Table 4), however, significant reduction was only found in the NMES-TA group (p = 0.028 for static spasticity and p = 0.025 for dynamic spasticity, respectively). Significant group differences in the dynamic spasticity during walking were noted between the NMES-TA and NMES-MG groups (p = 0.044). Muscle strength of ankle dorsiflexors was only significantly improved in the NMES-TA group after training (p = 0.012; Table 4). Significant group difference in the muscle strength of ankle dorsiflexors was mainly detected between the NMES-TA and the control groups (p = 0.009). Muscle strength of ankle plantarflexors was not significantly improved after training in all three groups, and no significant group differences in change scores were found.

**Table 4:** Muscle strength and spasticity before and after training in 3 groups.

	Control (N = 8)		NMES-TA (N = 8)		NMES-MG (N = 9)	
	Pre	Post	Pre	Post	Pre	Post
Ankle dorsiflexors strength (N)	89.9±11.4	86.2±10.6	76.5±14.0	109.1±14.6*	92.9±9.1	108.9±11.9
** **Change values (%)^a^		-2.3±13.7		38.5±39.4#		20.3±27.5
Ankle plantarflexors strength (N)	112.9±16.5	126.2±8.2	104.4±9.6	124.9±11.9	110.9±8.2	128.8±8.9
** **Change values (%)^a^		26.2±47.4		20.7±20.2		20.2±32.9
MAS(0–5)	1.9±0.4	1.5±0.1	2.4±0.3	1.5±0.3*	2.1±0.3	1.7±0.3
** **Change values (%)^a^		-2.2±54.4		-36.3±35.4		-18.4±31.1
Spasticity index	3.4±4.2	-2.1±3.1	9.8±2.5	-9.8±3.6*	7.2±2.8	3.8±3.8
** **Change values (%)^a^		-141.6±257.3		-303.1±176.8^╪^		-124.7±224.8

Abbreviations: MAS: modified Ashworth scale

^a^ Change values were calculated by subtracting the pre-training data (baseline) from the post-training data divided by the pre-training data and expressed as %

*, p<0.05 for intra-group difference

#, p<0.05 compared with control group

^╪^, p<0.05 compared with NMES-MG

After training, the maximum position of ankle dorsiflexion at heel strike did not significantly change in all three groups, and there were no significant group differences in change scores detected (Table 5). Compared to the control group, the NMES-TA group showed a significant improvement in ankle plantarflexion during push off (p = 0.015). For the CV of maximum position of ankle joint at heel strike and in push off, there were neither significant pre-post training differences in all three groups nor group differences in change score detected.

**Table 5:** The active range of motion of the ankle joint during gait before and after training in 3 groups.

	Control (N = 8)		NMES-TA (N = 8)		NMES-MG (N = 9)	
	Pre	Post	Pre	Post	Pre	Post
CV of ankle dorsiflexion at HS (%)	10.5±1.9	12.4±3.8	7.2±1.5	5.6±1.5	10.1±2.9	8.8±3.7
** **Change values (%)^a^		9.5±98.8		-21.6±45.0		-5.9±65.4
CV of ankle plantarflexion in push off (%)	34.4±15.8	21.3±7.1	19.9±4.9	29.6±7.7	42.9±12.9	22.1±5.4
** **Change values (%)^a^		-20.2±81.1		14.1±63.2		-19.7±85.2
MP of dorsiflexion at HS (degree)	-21.9±2.3	-25.2±1.7	-25.3±3.6	-24.1± 2.8	-21.6±2.9	-27.9±3.8
** **Change values (%)^a^		-30.9±70.5		8.5±56.2		-36.4±57.3
MP of plantarflexion in push off (degree)	-6.2±1.0	-10.3±1.1*	-10.3±1.7	-8.5±0.9	-10.1±1.9	-11.7±1.4
** **Change values (%)^a^		-52.4±24.4		3.6±38.7#		-22.7±36.8

Abbreviations: CV: coefficient of variation; HS: heel strike; MP: Maximum position

^a^ Change values were calculated by subtracting the pre-training data (baseline) from the post-training data divided by the pre-training data and expressed as %

*, p<0.05 for intra-group difference

#, p<0.05 compared with control group

## Discussion

For chronic stroke people with inadequate ankle control, our results found that a total of 21 sessions of NMES on ankle dorsiflexors resulted in increased step length, spatial gait symmetry, and active ankle plantarflexion during push off, together with decreased static and dynamic plantarflexors spasticity and increased dorsiflexors muscle strength. However, the NMES on plantarflexors could only improve the step length and temporal gait symmetry.

Although there was no significant improvement in gait velocity after either NMES training as indicated by change values, a previous study has shown that a real change of minimal improvements in gait velocity should be achieved by a 7.9% increase [21]. In this study, both NMES-TA and NMES-MG groups achieved the real change by a 30.6% increase and a 13.6% increase, respectively (only a 2.4% increase in the control group). The increased step length in both NMES groups is suggested to contribute to the increased gait velocity in our study. In addition, the relatively more increase in gait velocity in NMES-TA group may at least in part attribute to the improved dorsiflexors muscle strength and plantarflexors spasticity since these two improvements could account for 45% of the variance in gait velocity in patients with stroke [3].

The study showed that spatial asymmetry was significantly reduced in the NMES-TA group as compared with the control group. Also, it was noted that both static and dynamic plantarflexors spasticity decreased after intervention in NMES-TA group. Previous study noted that the spatial symmetry is strongly correlated with spasticity of ankle plantarflexors. Hsu et al. reported that static plantarflexors spasticity was the most important independent determinant of spatial gait asymmetry (R^2^ = 0.46) [22]. Our previous study further demonstrated that the dynamic plantarflexors spasticity could explain 53% of variance in spatial symmetry in people with stroke [3]. Therefore, NMES applied on the tibialis anterior could improve spatial symmetry efficiently in our participants which may be due to decreasing both static and dynamic plantarflexors spasticity.

On the other hand, the temporal symmetry was significantly improved only in the NMES-MG group after training. It has been shown that spasticity of ankle plantarflexors, motor function of the affected leg, position sense of ankle, and postural sway were correlated with temporal gait symmetry [22, 23]. However, the changes in motor function, sensation, and balance control were not measured, and thus whether these changes could be enhanced by NMES applied on the gastrocnemius are not known in this study. Future studies are encouraged to include different measurement aspects to elucidate the possible reasons for NMES effects on temporal gait symmetry.

Previous studies have shown that NMES could enhance muscle strength in chronic stroke individuals through increasing the number of activated motor units, the rate of activation, and synchronization of activation [24, 25]. Our data showed that muscle strength of ankle dorsiflexors was significantly improved after applying NMES on TA, however, muscle strength of ankle plantarflexors did not change significantly after applying NMES on MG. According to Bergquist et al. applying NMES over the nerve trunk would be more effective than over muscle belly because the former could result in muscle contractions with greater motor unit recruitment and less muscle fatigue [26]. Therefore, the application on nerve trunk instead of muscle belly is suggested to improve muscle strength of gastrocnemius by using NMES. In addition, we noted the participants in the NMES-MG group tolerated less intensity than those in the NMES-TA group. Such different tolerance in ES intensity may explain partly the different results in reducing the dynamic plantarflexor spasticity. It is known that activation of Golgi tendon organ (GTO) of a spastic muscle, which is part of the Ib inhibitory pathway, could decrease the spasticity [27]. Due to the high activation threshold of GTO, the intensity of ES used in the NMES-MG group in our study may not be sufficient to reduce spasticity of plantarflexors via the GTO firing. In addition to intensity, the frequency of ES used is another factor that needs to be considered. It has been reported that the frequency of ES should be greater than 100 Hz to induce neuromuscular junction fatigue or possible depletion of acetylcholine to reach the goal of relieving spasticity using NMES [11, 28–30]. It is possible that the frequency of 50 Hz used in the current study was not high enough to induce muscle fatigue or acetylcholine depletion to decrease spasticity of plantarflexors. However, the significant reduction of dynamic spasticity noted after the NMES applied on TA indicated the possible better strategy for clinical application.

Active ankle plantarflexion in push off, determined by ankle plantarflexion kinetics and kinematics, is a critical ankle motion for forward progression of the body during normal gait [9]. Our findings in the improved ankle plantarflexion kinematics during push off in the NMES-TA group could be due to the decreased static and dynamic spasticity of plantarflexors. Lamontagne et al. showed that a positive slope of dynamic spasticity index during stance phase of a gait cycle is related to a dominant velocity-sensitive muscle activation pattern, which is compatible with an increased excitation of the stretch reflex and a lower stretch reflex threshold [7]. It is likely that the hyperactivity of plantarflexors in its lengthening period from mid-stance to terminal stance compromises the kinematics of ankle joint and the efficiency of plantarflexors during push off. Therefore, reducing spasticity of ankle plantarflexors could enhance the active ankle control during push off.

Spasticity of the ankle joint was also found to be negatively correlated with antagonist voluntary contraction in hemiparetic individuals [31]. However, despite the increased muscle strength of ankle dorsiflexors and decreased spasticity of plantarflexors, active ankle dorsiflexion at heel strike was not significantly improved in the NMES-TA group after training. Such finding is in accordance with the study done by Yavuzer et al. [32]. Instead of using a heel contact that generates ankle plantarflexion motions at heel strike, spastic gait often had a forefoot contact with the floor which drives the heel toward the ground with dorsiflexion motions. Studies have shown such dorsiflexion motions during forefoot contact could induce abnormal dorsiflexion angular velocity that may result in a reflex activation of ankle plantarflexors at heel strike [33, 34]. This phenomenon may be related to the limited training effects on active ankle dorsiflexion at heel strike in the NMES-TA group observed in our study.

There are some limitations in the current study. First, significant differences of between- and within-group training effects may not be demonstrated due to small sample size. Second, it is unclear whether increasing intensity of NMES over ankle plantarflexors would alter the findings of the current study. Third, the therapist who conducted the training, was not blinded to group assignment; although unavoidable, this limitation may have introduced bias. Further investigations are needed to determine the optimal intensity of NMES on ankle dorsiflexors and plantarflexors on a larger sample population with double-blinded setting.

In conclusion, our findings suggest that applying NMES on ankle dorsiflexors with ambulation training might be an effective strategy for muscle strengthening and spasticity reducing to enhance ankle control during push off and gait performance. While applying NMES on ankle plantarflexors with ambulation training could result in favorable effects on temporal gait symmetry in chronic stroke individuals with inadequate ankle control.

## Supporting information

S1 File CONSORT checklist. (DOC)

S2 File Protocol submitted to IRB. (DOCX)

S3 File Study protocol_English main points. (DOCX)
