# Supplementary material for: Effects of neuromuscular electrical stimulation on gait performance in chronic stroke with inadequate ankle control - A randomized controlled trial
Source: PLoS One. 2018 Dec 10;13(12):e0208609. doi: 10.1371/journal.pone.0208609 (PMC6287810; doi:10.1371/journal.pone.0208609)
Supplement: S2 File — (DOCX) [file pone.0208609.s002.docx]

**研究計畫書**

**神經肌肉電刺激訓練對慢性中風患者踝關節控制與步態之效果**

**The effects of neuromuscular electrical stimulation on ankle control and gait performance in individuals with chronic stroke：A randomized controlled trial**

試驗主持人：黃士峯 醫師

共同主持人：王瑞瑤 教授

第二版

簽名： 日期：102.7.10

**目錄**

[中文摘要 3](#_Toc282980591)

[英文摘要 5](#_Toc282980592)

[第一章 簡介 7](#_Toc282980593)

[第一節 研究背景與動機 7](#_Toc282980594)

[第二節 研究目的 7](#_Toc282980595)

[第三節 研究假設 8](#_Toc282980596)

[第四節 重要性 8](#_Toc282980597)

[第二章 文獻回顧 9](#_Toc282980598)

[第一節 中風步態的缺失 9](#_Toc282980599)

[第二節　腳踝損傷對動作控制與步態的影響 9](#_Toc282980600)

[第三節　神經肌肉電刺激對於中風患者改善肌力、痙攣與步態的功效 10](#_Toc282980601)

[第三章 研究方法 12](#_Toc282980603)

[第一節 研究設計與研究架構 12](#_Toc282980604)

[第二節　研究方法 12](#_Toc282980605)

[參考文獻 20](#_Toc282980606)

中文摘要

**背景：**中風是造成病人慢性失能的主要原因之一。行走上的障礙被認為是中風後最常出現的功能限制之一。中風後行走功能上的障礙常見的特徵有因下肢肌肉無力、不良動作控制和痙攣所造成之步行速度降低與步態不對稱。而在腳踝處的蹠屈肌與背屈肌的無力、蹠屈肌的痙攣都被認為可能會顯著影響中風病人之腳踝控制與步態功能的表現。目前用以改善腳踝關節動作缺失的方式有傳統肌力訓練、高肌肉張力改善策略、儀器治療以及步態行走訓練。其中，神經肌肉電刺激治療為一種中風復健中臨床常見於結合其他介入的治療方式，過去的學者們認為神經肌肉電刺激在中風病人身上不僅能改善肌力也能降低痙攣肌的痙攣程度。雖然過去的研究中，神經肌肉電刺激被認為對改善肌肉的肌力與痙攣有正向的效果，但大多數的研究都是著重上肢部分，在下肢的研究則較少且結果上仍有歧異。再者，上述的研究中皆針對靜態痙攣的改善，而對影響步態的動態痙攣及踝關節控制則少有探討。此外，大部分的研究中，都低估了神經肌肉電刺激在改善腳踝蹠屈肌肌力對於中風病人改善步態表現時所扮演的角色，因此本研究的目的為探討應用於不同肌肉群的神經肌肉電刺激治療對於中風患者腳踝控制與步態之效果。

**方法：**45位腳踝控制不足的慢性期中風患者將被隨機分派至控制組(15位)、神經肌肉電刺激-踝背屈肌組(15位)與神經肌肉電刺激-踝蹠屈肌組(15位)。神經肌肉電刺激-踝背屈肌組將接受應用於腳踝背屈肌之神經肌肉電刺激20分鐘；神經肌肉電刺激-踝蹠屈肌組將接受應用於腳踝蹠屈肌之神經肌肉電刺激20分鐘；控制組將接受20分鐘傳統物理治療包括踝關節被動關節活動運動、被動牽拉運動及主動-協助關節活動(Active- assistive range of motion)運動。隨後，三組均再接受強調足踝控制的行走訓練15分鐘。三組受試者均接受一週三次，為期七週的介入，共計二十一次。在介入前及完成二十一次治療介入後會各測量一次足踝痙攣程度、足踝肌力、腳踝控制與步態表現。步態表現利用步態分析儀(GaitRite system)測量行走之速度、步頻、步長，空間與時間之不對稱性。腳踝蹠屈肌的痙攣測量以Modified Ashworth score、H 反射與步行時的動態痙攣程度來表示。除此之外，足踝關節肌力的測量會使用手握式肌力測量儀(handheld dynamometer)測量踝關節蹠屈肌與背屈肌肌力，以及在步態週期中使用BIOPAC 16頻道、資料擷取/信號分析系統收取踝關節背屈肌於著地期與蹠屈肌於推進期的肌電圖活動。腳踝控制的測量則使用步態週期中著地期之踝關節主動背屈角度與推進期之踝關節主動蹠屈角度，利用分析變異系數(Coefficient of variances)表示腳踝控制的能力。**統計分析：**基準值的類別資料與連續性資料將分別使用卡方檢定與單因子變異數分析。治療前後的效果差異會使用重複測量雙因子多變量變異數分析，以Tukey post hoc test為事後檢定。所有顯著水平訂為p<0.05。

**關鍵字：**神經肌肉電刺激、腳踝控制、動態痙攣、步態表現、中風

英文摘要

**Background:** Stroke is the main cause of the chronic physical disability. After stroke, walking dysfunction appears to be one of the most frequently reported limitations. The common features of walking dysfunction after stroke are decreased gait velocity and asymmetry gait pattern, which may be resulted from spasticity, muscle weakness or poor motor control in the lower extremity. Weakness in ankle dorsiflexors and plantarflexors and spasticity in plantarflexors were reported to affect the ankle control and the gait performance in patients with stroke. Interventions administered to improve above mentioned ankle impairments including muscle training, strategies for managing hypertonia, modality and locomotion training. The neuromuscular electric stimulation (NMES), which has been demonstrated not only to improve muscle strength but also decrease spasticity via reciprocal inhibition, is a common clinical modality used to combine with other interventions for stoke rehabilitation. It seems NMES may exert positive effects on motor impairments in stroke patients. However, such positive effects are not well documented in ankle joint and the underlying mechanisms are still unclear. Furthermore, the applying of NMES was usually on the ankle dorsiflexor, which might underestimate the effect of NMES applied on ankle plantarflexor for strengthening muscle power to improve ankle control and gait performance. The purpose of this study is to investigate the effects of NMES applied to different muscle groups on ankle control and gait performance in chronic stroke subjects.

**Method:** 45 chronic stroke subjects with inadequate ankle control will be recruited to this study. Subjects will be randomly assigned to one of the 3 groups, the control, NMES on ankle dorsiflexors and NMES on plantarflexors group. Subjects in the NMES on ankle dorsiflexors group will receive 20 minutes of NMES on ankle dorsiflexors, followed by 15 minutes of ambulation training. Subjects in the NMES on plantarflexors group will receive 20 minutes of NMES on ankle plantarflexors, followed by 15 minutes of ambulation training. The control group will receive 20 minutes of traditional physical therapy, including passive range of motion exercise, stretch exercise and active-assisted range of motion exercise of ankle, followed by 15 minutes of ambulation training. The treatment session will be carried out 3 times a week for 7 weeks.

**Outcome measures:** The active ROM of ankle dorsiflexion at heel strike and active ROM of plantarflexion at push off during gait will be measured to indicate the ability of ankle control. Gait performance including speed, cadence, step length, and spatial and temporal symmetry will be measured by GaitRite system. The plantarflexors spasticity will be indicated by modified Ashworth score, H reflex and dynamic spasticity index during gait. The strength of dorsiflexors and plantarflexors will also be measured by handheld dynamometer. The EMG activity of dorsiflexors and plantarflexors will measured in different phase during gait by BIOPAC Data Acquisition System.

**Statistical analysis:** The one-way ANOVA and chi-square test will be used to compare the demographic and baseline data of the 3 groups. The 2-way ANOVA with repeated measures will be used for between group comparisons with Tukey post hoc test. The statistical significance is set at p < .05.

**Keywords**:

Neuromuscular electrical stimulation, ankle control, dynamic spasticity, gait performance, stroke

第一章 簡介

**第一節 研究背景與動機**

中風是造成成年人失能主要原因之一[^1^](#_ENREF_1)，而儘管這些病人接受了相關的復健，中風後仍會殘存行走功能缺失的問題，進而造成患者在社區中無法有效地行走[^2^](#_ENREF_2)。常見中風後行走步態缺失包括步行速度下降與步態不對稱[^3-6^](#_ENREF_3)，有許多學者提出不同原因會造成這些缺失，如肌力下降、動作控制不良與痙攣[^7-9^](#_ENREF_7)。其中，腳踝蹠屈肌與背屈肌肌力下降與蹠屈肌痙攣被認為會影響腳踝控制，並進一步的造成中風病人在步態的缺失[^5^](#_ENREF_5)^,^[^10^](#_ENREF_10)^,^[^11^](#_ENREF_11)。除此之外，Lin學者等人在2006年發現，行走時蹠屈肌所產生的動態痙攣是影響步態對稱性的一個決定性因素，並且在步行速度上也扮演關鍵性的角色[^11^](#_ENREF_11)。

目前用以改善上述腳踝關節動作損傷的方式有傳統肌力訓練、高肌肉張力改善策略、儀器治療以及步態行走訓練。其中，在神經肌肉電刺激治療的研究中，學者們認為神經肌肉電刺激不僅能改善肌力也能降低痙攣肌的痙攣程度[^12-22^](#_ENREF_12)。雖然過去的研究中，神經肌肉電刺激被認為對改善肌肉的痙攣有正向的效果，但大多數的研究都是著重上肢部分[^15^](#_ENREF_15)^,^[^17^](#_ENREF_17)^,^[^19^](#_ENREF_19)^,^[^23^](#_ENREF_23)，在下肢的研究則較少且結果上仍有歧異[^12-14^](#_ENREF_12)^,^[^16^](#_ENREF_16)^,^[^18^](#_ENREF_18)^,^[^20^](#_ENREF_20)。再者，上述的研究中皆針對靜態痙攣的改善[^12-14^](#_ENREF_12)^,^[^18^](#_ENREF_18)^,^[^20^](#_ENREF_20)，而對影響步態的動態痙攣則少有探討。此外，大部分的研究中，多低估了神經肌肉電刺激在改善腳踝蹠屈肌肌力對於中風病人改善步態表現時所扮演的角色[^12^](#_ENREF_12)^,^[^14^](#_ENREF_14)^,^[^18^](#_ENREF_18)^,^[^20^](#_ENREF_20)，因此本研究的目的為探討應用於踝蹠屈肌或踝背屈肌之神經肌肉電刺激治療對於中風患者腳踝控制與步態之效果。

**第二節 研究目的**

本研究目的為探討應用於踝蹠屈肌或踝背屈肌之神經肌肉電刺激治療對於中風患者腳踝控制與步態之效果。

**第三節 研究假設**

本研究的假設為慢性中風患者接受踝關節背屈肌之神經肌肉電刺激訓練較接受踝關節蹠屈肌之神經肌肉電刺激訓練在改善踝關節控制與步態上有顯著的差異；除此之外，有接受神經肌肉電刺激之患者在改善踝關節控制與步態上則較接受傳統物理治療為明顯。

**第四節 重要性**

由過去文獻顯示，中風患者可能會因為踝關節肌肉痙攣與肌力下降而造成行走能力上的缺失，但尚無有效的改善方式。而已被諸多研究認同的可改善中風患者肌肉痙攣的電刺激治療，仍然有許多限制與結果上的不一致。因此尋找一個有效的神經肌肉電刺激應用方式以改善中風患者肌肉痙攣與肌力下降的問題，並建立與步態的關聯性是非常重要的。

第二篇 文獻回顧

**第一節 中風步態的缺失**

典型的中風病人步態上的缺失包括了在站立期降低或喪失膝屈曲(knee flexion)動作，在擺盪期和著地初期喪失踝背屈(ankle dorsiflexion)動作與在站立末期缺乏踝蹠屈(ankle plantarflexion)動作^[24](#_ENREF_24)^。除此之外還包括行走速度下降、步態不對稱與軀幹傾向健側肢體的情形。而過去學者們認為諸如下肢肌力下降、蹠屈肌的動作功能控制不良與蹠屈肌的痙攣皆為可能造成此些步態的缺失[^6^](#_ENREF_6)。在2001年Lamontage學者的研究中，提出了在步行時測量動態痙攣的方式，並發現，中風病人在步態中的站立期，蹠屈肌會出現與健康人不同的肌肉活化方式，表現出增加的牽拉反射，即痙攣，而此一現象會影響到病人的行走功能，例如步行速度[^25^](#_ENREF_25)。

對於中風病人而言，有限恢復的步行功能被認為是造成失能的主要原因之一[^1^](#_ENREF_1)，因為這些步行上的障礙常常侷限了病人的活動，並且造成病人安全上的問題[^26^](#_ENREF_26)，因此步行功能的恢復被認為是最主要的復健目標之一。

**第二節 腳踝損傷對動作控制與步態的影響**

腳踝處的損傷在過去被認為可能會顯著影響慢性期中風病人步態的表現[^5^](#_ENREF_5)^,^[^10^](#_ENREF_10)^,^[^11^](#_ENREF_11)。腳踝處的損傷包括了背屈肌與蹠屈肌的無力、蹠屈肌的痙攣等，而這些損傷也被認為會進一步影響到病人腳踝控制的能力。Hsu等學者在2003年的研究中，提出了不同程度的蹠屈肌痙攣是決定輕度到中度中風病人步態不對稱性的最關鍵性因子[^5^](#_ENREF_5)。而Lamontage等學者利用了動態痙攣的概念，發現蹠屈肌動態痙攣會影響到中風病人的行走速度，為造成不良步行表現的因素之一[^25^](#_ENREF_25)。之後，Lin等學者在2006年的研究中，利用相同的概念也發現對有外出行走能力的中風病人，蹠屈肌動態痙攣程度是決定步態空間上不對稱性的唯一決定因子，且呈中度正相關。另外，蹠屈肌的動態痙攣與步行速度則呈中度負相關。除了蹠屈肌的痙攣會影響到病人的步態外，腳踝處的肌肉的控制也被認為是影響步態表現的因素之一。在Lin等學者的研究中，也指出腳踝背屈肌肌力是決定步行速度與步態時間上不對稱性的重要因素，且呈中度正相關，其與時間上的不對稱性則呈中度負相關。除此之外，腳踝處蹠屈肌肌力也與步行速度呈現中度正相關性[^11^](#_ENREF_11)。Kim等學者也同樣支持這個觀點，發現居住在社區的中風病人其髖屈肌、膝屈肌與蹠屈肌主動等速力矩與步行速度有顯著相關性，而其中又以蹠屈肌呈現高度相關性，更發現僅蹠屈肌力矩就能解釋步行速度72%的變異度^[10](#_ENREF_10)^。因此由過去的研究中，我們了解腳踝處的損傷所造成的控制不良可能會影響步態的表現。而目前常見處理上述腳踝關節損傷的方式，包含了傳統肌力訓練、高肌肉張力改善策略、儀器治療以及步態行走訓練[^21^](#_ENREF_21)^,^[^27^](#_ENREF_27)^,^[^28^](#_ENREF_28)。

**第三節 神經肌肉電刺激對於中風患者改善肌力、痙攣與步態的功效**

神經肌肉電刺激在過去被認為可改善中風病人動作功能。2006年，Barth等學者發現使用神經肌肉電刺激四週可改善慢性中風病人步行速度、步頻與步長[^29^](#_ENREF_29)。除此之外，神經肌肉電刺激也被認為可以增加肌力與改善痙攣肌的痙攣程度。在2008年Shin等學者針對中風病人之研究結果顯示，接受10週之上肢伸指總肌(extensor digitorum communis)的電刺激，可以誘發動作功能回復與大腦皮質變化，同時伴隨上肢伸指總肌肌力的改善[^19^](#_ENREF_19)。而在Cauraugh等學者將11位中風病人隨機分派到肌電誘發電刺激組與控制組，肌電誘發電刺激組的病人接受2週12回合的腕伸肌與指伸肌的電刺激後，其在平均力量的輸出與控制組呈現了顯著的差異性[^15^](#_ENREF_15)。根據過去研究的結果，神經肌肉電刺激有改善肌力的效果，而Nelson學者認為這可能與電刺激增加潛在可反應的動作單元或增加頻率，使徵召的動作單元活化速率的最大化有關[^27^](#_ENREF_27)。除了改善肌力的效果外，神經肌肉電刺激也同樣被認為可降低肌肉的痙攣程度，Lin等學者認為3週的神經肌肉電刺激的介入會增進中風病人上肢動作功能的恢復，並改善痙攣程度[^17^](#_ENREF_17)。而Mesci等學者則發現踝關節背屈肌經神經肌肉電刺激四週後，慢性期中風病人在踝關節背屈角度、蹠屈肌痙攣程度與下肢功能的改善都與未接受介入的控制組有顯著的差異[^18^](#_ENREF_18)。而神經肌肉電刺激改善痙攣肌肌肉痙攣的機制可能為電刺激刺激到拮抗肌之Ia訊息輸入神經，進而活化了在脊髓內的Ia聯絡神經元，並且經由交互抑制作用(reciprocal inhibition)，將低了痙攣肌興奮程度^[27](#_ENREF_27)^。然而，在過去的研究中也有出現相反的結果，如Gunes等學者提出了4週的神經肌肉電刺激在中風病人身上，對於下肢的動作恢復與步態上並沒有比一般傳統的復健治療效果好^[30](#_ENREF_30)^。在2008年，Bakhtiary等學者發現介入20天神經肌肉電刺激治療於中風病人，在以H/M ratio所測定之蹠屈肌痙攣上，比起Bobath介入手法，並沒有更顯著的改善效果[^20^](#_ENREF_20)。Embrey等學者於2012年也指出中風病人在訓練步態中使用3個月的活化踝關節背屈肌與蹠屈肌的功能性電刺激(Functional electrical stimulation)介入，對於蹠屈肌的肌力與痙攣情形，沒有顯著得改善效果^[13](#_ENREF_13)^。並且，回顧過去的研究，大多數神經肌肉電刺激或功能性電刺激的探討，都以降低靜態痙攣程度來當作結果，與步態表現較有關係之動態痙攣則少有提及。除此之外，多數研究中也都未提及其介入方式是否對蹠屈肌肌力的改變有所影響[^12^](#_ENREF_12)^,^[^14^](#_ENREF_14)^,^[^18^](#_ENREF_18)^,^[^20^](#_ENREF_20)。其中，Embrey等學者的研究中，雖有測量蹠屈肌肌力的變化情形，卻發現功能性電刺激對於增強蹠屈肌肌力並無顯著影響[^13^](#_ENREF_13)。因此不論是在中風病人下肢痙攣，肌力或步態功能上，神經肌肉電刺激的效果，仍需進一步作全面的探討。

第三章 研究方法

**第一節 研究設計與研究架構**

本研究為單盲隨機分派之臨床試驗（single-blinded, randomized controlled trial），評估者不知道受測者分配的組別。在受試者參與本研究前，會對其先說明所有研究的流程，受試者可自行選擇與否參加。同意參加的受試者在簽署同意書後將被隨機分派至控制組、神經肌肉電刺激-踝背屈肌組與神經肌肉電刺激-踝蹠屈肌組。隨後會進行基本資料收集及介入前的評估(pretreatment evaluation)。神經肌肉電刺激-踝背屈肌組接受踝關節背屈肌之神經肌肉電刺激治療20分鐘，神經肌肉電刺激-踝蹠屈肌組接受踝關節蹠屈肌之神經肌肉電刺激治療20分鐘，而控制組接受20分鐘傳統物理治療包括踝關節被動關節活動，被動牽拉運動及主動-協助關節活動。之後，三組均再接受以語言提示(verbal cue)強調足踝控制的行走訓練15分鐘。治療頻率每週三次，共計七週，完成七週介入後再進行介入後的評估(post-treatment evaluation)

**第二節 研究方法**

**一、研究對象**

本研究預計招募45位中風後患者，收案標準如下：（一）屬於第一次中風，並且中風至少6個月；（二）步態週期中，病患著地期所執行之主動踝關節背屈角度小於5度；（三）步態週期中，病患於推進期所執行之主動踝關節蹠屈角度小於10度；（四）病人不論有無使用輔具能行走至少6公尺距離；（五）患側脛前肌與腓腸肌具大於5微伏特(>5 µV)之肌電訊號；(六)患側踝關節能維持在正中姿勢(neural position)；排案標準如下：（一）感覺功能損傷或喪失；（二）其它會影響到行走功能的骨科性與神經性問題；（三）電療儀器之相關禁忌症，例如靜脈血栓炎，心律節律器、惡性腫瘤等；(四)認知能力(簡易智能量表小於23分)或溝通能力不足無法參與本研究。

**二、測試程序**

受試者同意參與本研究後，將記錄其基本資料，如：性別、年齡、損傷側大腦、受傷後時間、病人所使用的輔具與之前所接受過的介入以及用藥等。

**三、評估項目**

本研究治療前後之評估項目包括痙攣程度、步態表現、肌力與踝關節控制。痙攣程度會檢測H反射（沃夫曼反射，Hoffmann reflex）、徒手檢測(Modified Ashworth score)與動態痙攣指數，目標肌肉為踝關節蹠屈肌。肌力的測量會使用手握式肌力測量儀(handheld dynamometer)，目標肌肉為踝關節蹠屈肌與背屈肌。步態週期中肌力的測量會使用BIOPAC 16頻道、資料擷取/信號分析系統收取踝關節背屈肌於著地期與蹠屈肌於推進期的肌電圖活動。步態表現會以步態分析儀(GAITRite system)來做測量，收取步態表現的相關參數。踝關節控制的測量則使用步態週期中著地期之踝關節主動背屈角度與推進期之踝關節主動蹠屈角度，利用分析變異系數(Coefficient of variances)來表示。

**(一)痙攣程度**

**1.徒手檢側**

臨床上常使用修正版艾許沃斯量表評分痙攣程度，在平躺下，髖關節與膝關節保持在0度的姿勢下檢測。利用快速牽拉目標肌肉時所得到的阻力變化來評分。0分代表肌肉張力沒有不正常的增強；1分代表張力些微的增加，且關節活動度末端才會出現輕度的阻力；2分代表肌肉張力些微的增強，小於關節活動度一半以內的範圍會出現輕度的阻力；3分代表肌肉張力明顯的增加，整個關節活動度的範圍內有明顯的張力增加，但肢體是容易被移動的；4分代表肌肉張力很大，要被動地移動關節是很困難的；5分代表關節固定在彎曲或伸直的位置。於患側重複施測三次^[31](#_ENREF_31)^。

**2.H反射**

H反射的大小代表下肢脊髓內興奮性的變化，當抑制性訊號減弱，H反射訊號將會變大。會使用肌電計（Medelec EMG/EP Diagnostic Instruments, Synergy, Oxford Instruments Medical Ltd., UK）偵測訊號，在蹠屈肌肌腹貼上電極片，以收取H反射的訊號，受試者會趴在治療床上，在膝蓋下方找到合適刺激位置後將電極固定，電刺激後脛神經（posterior tibial nerve）。將會調整刺激強度以測得最大的H反射和M反應的最大振幅，並重複5-8次刺激，H反射刺激間隔大於10秒，M反應刺激間隔大於10秒。電刺激時間為0.5ms，訊號也會經過處理（bandpass filter, 20 Hz to 1 kHz），測量連續兩個波峰之間（peak-to-peak）的大小，H/M比值為最大H反射之振幅除以最大M反應之振幅，代表目標肌肉痙攣的情形，本研究將以此值代表肌肉痙攣的程度[^32^](#_ENREF_32)^,^[^33^](#_ENREF_33)。

**3.動態痙攣指數**

痙攣指數為可在步態中評估動態痙攣程度。痙攣指數的定義是肌電活動與內側腓腸肌在步態週期之站立期肌肉伸長的速度二者之間的斜率。正斜率代表動作模式對於速度是較敏感的，對於痙攣而言，此斜率可視為反射增加的一種情形[^25^](#_ENREF_25)。因為不是每個週期都是正斜率，因此僅選擇正斜率的資料進行分析。內側腓腸肌的肌電活動訊號是使用11公分的Ag-AgCl電極。使用前會以酒精清潔皮膚，若有必要也會刮除部分毛髮以降低阻抗。電極擺放的位置在肌肉的運動點，大約位於膕窩下方五指幅寬和中線往內側兩公分的位置。肌電圖訊號會放大(放大率為375)並透過模數轉換(analog-to-digital convert)儲存至個人電腦。

將電子量角器(Twin Axis Goniometer, SG110, Biometrics Ltd., UK)放置在雙側膝及踝關節評估步態週期中角度的變化。踝關節電子量角器的軸心位於外踝(lateral malleolus)，一端會沿著外踝和腓骨頭部(fibular head)之間連線放置，另一端則延著外踝和第五蹠骨頭部(fifth metatarsal head)間的連線放置。膝關節電子量角器的軸心位於股骨外上髁(lateral epicondyle of the femur)和大轉子(greater trochanter)之間的連線放置，另一端則沿著股骨外上髁和外踝之間的連線放置。最後會在腳趾和腳跟位置放置足踏開關(footswitch)用以偵測步態週期中的站立期或擺盪期。

所有的訊號會由AcqKnowledge software第3.7.5版和BIOPAC 16頻道、資料擷取/信號分析系統(BIOPAC Data Acquisition System, MP150WSW, BIOPAC system, Inc, USA)以1000 Hz的選取頻率記錄並儲存。肌電圖的共模拒絕比為104 dB；模數轉換比為86 dB；解析度為16位元；增益為2000。肌電圖分析的流程參考Lamontagne等學者之研究[^25^](#_ENREF_25)。內側腓腸肌在站立期肌肉伸長的肌電圖會經過標準化的程序呈現站立時期的最大值。而肌肉伸長的速度則會參考Winter和Scott學者的模型。最後的斜率、痙攣指數將會計算出[^34^](#_ENREF_34)。

**(二)步態表現**

使用步態分析儀(GAITRite system)收取步態表現的參數。步態分析儀為長3.66公尺、寬0.61公尺之地毯走道，走道上有六個感應板，內有13824個感應器，當受試者在行走時，走道內的感應器會收集受試者的步態表現。此分析儀會連結到個人電腦上，並顯示出所收集到的資料，資料內容包括：步行速度(Speed)、步頻(Cadence)、左右腳步長(step length)、左右腳步寬(support base width)、左右腳單腳支撐時間(single support time)、左右腳雙腳支撐時間 (double support time)等步態參數值[^11^](#_ENREF_11)^,^[^35-37^](#_ENREF_35)。受測者在執行步行功能可使用行走輔具。除此之外會利用所得兩腳之踏步長，計算出步態上空間的不對稱(Spatial asymmetry)，計算公式如下：1- $\frac{患側腳踏步長}{健側腳踏步長}$；而利用兩腳單腳承重時間可計算出步態上時間上的不對稱(Temporal asymmetry)，計算公式如下：1- $\frac{患側腳單腳承重時間}{健側腳單腳承重時間}$。此研究方法為本實驗室收取步態表現相關參數的方法之一[^11^](#_ENREF_11)。

**(三)肌力**

**1.靜態肌力**

使用手握式肌力測量器(handheld dynamometer)在平躺姿勢下測量，將測量儀垂直放置在目標肌肉的肌腹上(脛前肌、腓腸肌)，脛前肌測量時下肢的擺位為髖關節與膝關節呈現90度，而測量腓腸肌時，下肢擺位為髖關節與膝關節皆呈現0度。肌力測量方式為：請受試者做出三次最大力量之等長收縮，每次收縮時間為5秒鐘，每次收縮間隔時間為5至10秒，使肌肉以最佳狀態表現出最大收縮值，並避免肌肉產生過度疲憊之現象。測試程序為先測試健側下肢，再測試患側下肢，測量結果為3次數值的平均值^[16](#_ENREF_16)^。

**2. 動態肌力**

本研究動態肌力以步態週期中踝關節背屈肌於著地期與蹠屈肌於推進期之肌電圖活動訊號來表示。背屈肌的目標肌肉為脛前肌，蹠屈肌的目標肌肉為內側腓腸肌。脛前肌與內側腓腸肌的肌電圖活動訊號是使用11公分的Ag-AgCl電極。使用前會以酒精清潔皮膚，若有必要也會刮除部分毛髮以降低阻抗。電極擺放的位置皆在肌肉的運動點上，內側腓腸肌之位置約位於膕窩下方五指幅寬和中線往內側兩公分處，脛前肌之位置約位於腓骨頭與內踝連線的三分之一處。肌電圖訊號會放大(放大率為375)並透過模數轉換(analog-to-digital convert)儲存至個人電腦。

兩條肌肉之訊號會由AcqKnowledge software第3.7.5版和BIOPAC 16頻道.資料擷取/信號分析系統(BIOPAC Data Acquisition System, MP150WSW, BIOPAC system, Inc, USA)以1000 Hz的選取頻率記錄並儲存。肌電圖的共模拒絕比為104 dB；模數轉換比為86 dB；解析度為16位元；增益為2000。

**(四)腳踝控制能力**

本研究以步態週期中著地期之踝關節主動背屈角度與推進期之踝關節主動蹠屈角度，利用分析變異系數(Coefficient of variances)表示腳踝控制的能力。在Cheng學者2010年的研究中認為，當踝關節背屈之主動關節角度變異系數變小時，代表受試者每次在著地期所表現的腳踝背屈之主動關節角度的變化變小，可代表腳踝控制的能力增加[^16^](#_ENREF_16)。

**四、神經肌肉電刺激**

將使用肌電誘發之神經肌肉電刺激(生理回饋合併電刺激器，Myomed 932；Enraf Nonius, The Netherlands)在病人坐姿下，分別收取與訓練之目標肌肉的肌電訊號，並且當所收取的肌電訊號超過設定的閾值後，會誘發啟動一個給予目標肌肉的電刺激，而此神經肌肉電刺激為肌電誘發之神經肌肉電刺激(EMG-trigger NMES)。在兩組神經肌肉電刺激組，應用的位置分別為脛前肌與腓腸肌。脛前肌之電擊片放置方式為兩片1.5X2公分表面電擊片分別置於脛前肌之運動點及肌腹上。腓腸肌之電擊片放置方式則是將相同尺寸的兩片表面電擊片，皆置於腓腸肌肌腹上。除此之外，直徑1.5X2公分的參考電極片則放置於兩表面電極片的遠端。電刺激的設定參數：波型：雙相波；波寬：0.2毫秒；波頻：50赫茲；電刺激強度：0到50毫安培(視病人情況而定，必須使病人踝關節作出最大範圍的背屈動作)；肌電訊號偵測閾值：5到80微安培(視病人實際情況而定)；電刺激時間：20分鐘。神經肌肉電刺激組病人會接受神經肌肉電刺激治療，每週三次，連續接受七週，共計21次治療。

控制組則接受20分鐘傳統物理治療包括踝關節被動關節活動，被動牽拉運動及主動-協助關節活動，每週三次，連續七週，共計21次。

**六、統計分析**

研究資料以SPSS(Version 20.0)套裝軟體進行分析。統計值的顯著水平定在0.05，所有分析的數值會以平均值±標準差(Mean±SD)的方式呈現。各組受測者治療前的基本資料和評估項目將以描述性統計方式呈現，包括性別、受傷側大腦、年齡、受傷後的時間、痙攣程度、步態等資料。組間以卡方檢定(Chi-square test)比較類別性資料，用單因子變異數分析(One-way Analysis of Variance )比較連續性資料。以重複測量兩因子變異數分析(Two-way Analysis of Variance with Repeated measures) 比較組間與組內前後測之測量值，事後檢定的部分會以Tukey post hoc test分析。顯著水平訂為p<0.05。

參考文獻

**1.** Whitall J. Stroke rehabilitation research: time to answer more specific question? *Neurorehabilitation and Neural Repair.* 2004;18(1):3-8.

**2.** Bohannon RW, Andrews AW, Smith MB. Rehabilitation goals of patients with hemiplegia. *International Journal of Rehabilitation Research.* 1988;11(2):181-184.

**3.** Patterson KK, Gage WH, Brooks D, Black SE, McIlroy WE. Evaluation of gait symmetry after stroke: a comparison of current methods and recommendations for standardization. *Gait & Posture.* 2010;31(2):241-246.

**4.** Ozgirgin N, Bolukbasi N, Beyazova M, Orkun S. Kinematic gait analysis in hemiplegic patients. *Scandinavian Journal of Rehabilitation Medicine.* 1993;25(2):51-55.

**5.** Hsu AL, Tang PF, Jan MH. Analysis of impairments influencing gait velocity and asymmetry of hemiplegic patients after mild to moderate stroke. *Archives of Physical Medicine and Rehabilitation.* 2003;84(8):1185-1193.

**6.** Titianova EB, Tarkka IM. Asymmetry in walking performance and postural sway in patients with chronic unilateral cerebral infarction. *Journal of Rehabilitation Research and Development.* 1995;32(3):236-244.

**7.** De Quervain IA, Simon SR, Leurgans S, Pease WS, McAllister D. Gait pattern in the early recovery period after stroke. *The Journal of Bone and Joint Surgery. American volume.* 1996;78(10):1506-1514.

**8.** Lehmann JF, Condon SM, Price R, deLateur BJ. Gait abnormalities in hemiplegia: their correction by ankle-foot orthoses. *Archives of Physical Medicine and Rehabilitation.* 1987;68(11):763-771.

**9.** Tyson SF. Trunk kinematics in hemiplegic gait and the effect of walking aids. *Clinical Rehabilitation.* 1999;13(4):295-300.

**10.** Kim CM, Eng JJ. The relationship of lower-extremity muscle torque to locomotor performance in people with stroke. *Physical Therapy.* 2003;83(1):49-57.

**11.** Lin PY, Yang YR, Cheng SJ, Wang RY. The relation between ankle impairments and gait velocity and symmetry in people with stroke. *Archives of Physical Medicine and Rehabilitation.* 2006;87(4):562-568.

**12.** Sabut SK, Sikdar C, Kumar R, Mahadevappa M. Functional electrical stimulation of dorsiflexor muscle: effects on dorsiflexor strength, plantarflexor spasticity, and motor recovery in stroke patients. *NeuroRehabilitation.* 2011;29(4):393-400.

**13.** Embrey DG, Holtz SL, Alon G, Brandsma BA, McCoy SW. Functional electrical stimulation to dorsiflexors and plantar flexors during gait to improve walking in adults with chronic hemiplegia. *Archives of Physical Medicine and Rehabilitation.* 2010;91(5):687-696.

**14.** Sabut SK, Sikdar C, Mondal R, Kumar R, Mahadevappa M. Restoration of gait and motor recovery by functional electrical stimulation therapy in persons with stroke. *Disability and Rehabilitation.* 2010;32(19):1594-1603.

**15.** Cauraugh J, Light K, Kim S, Thigpen M, Behrman A. Chronic motor dysfunction after stroke: recovering wrist and finger extension by electromyography-triggered neuromuscular stimulation. *Stroke.* 2000;31(6):1360-1364.

**16.** Cheng JS, Yang YR, Cheng SJ, Lin PY, Wang RY. Effects of combining electric stimulation with active ankle dorsiflexion while standing on a rocker board: a pilot study for subjects with spastic foot after stroke. *Archives of Physical Medicine and Rehabilitation.* 2010;91(4):505-512.

**17.** Lin Z, Yan T. Long-term effectiveness of neuromuscular electrical stimulation for promoting motor recovery of the upper extremity after stroke. *Journal of Rehabilitation Medicine.* 2011;43(6):506-510.

**18.** Mesci N, Ozdemir F, Kabayel DD, Tokuc B. The effects of neuromuscular electrical stimulation on clinical improvement in hemiplegic lower extremity rehabilitation in chronic stroke: a single-blind, randomised, controlled trial. *Disability and Rehabilitation.* 2009;31(24):2047-2054.

**19.** Shin HK, Cho SH, Jeon HS, et al. Cortical effect and functional recovery by the electromyography-triggered neuromuscular stimulation in chronic stroke patients. *Neuroscience Letters.* 19 2008;442(3):174-179.

**20.** Bakhtiary AH, Fatemy E. Does electrical stimulation reduce spasticity after stroke? A randomized controlled study. *Clinical Rehabilitation.* 2008;22(5):418-425.

**21.** Bhakta BB. Management of spasticity in stroke. *British Medical Bulletin.* 2000;56(2):476-485.

**22.** Hsu A, Tang P, Jan M. Analysis of impairments influencing gait velocity and asymmetry of hemiplegic patients after mild to moderate stroke. *Archives of Physical Medicine and Rehabilitation.* 2003;84(8):1185-1193.

**23.** Theilig S, Podubecka J, Bösl K, Wiederer R, Nowak DA. Functional neuromuscular stimulation to improve severe hand dysfunction after stroke: Does inhibitory rTMS enhance therapeutic efficiency? *Experimental Neurology.* 2011;230(1):149-155.

**24.** Bensoussan L, Mesure S, Viton JM, Delarque A. Kinematic and kinetic asymmetries in hemiplegic patients' gait initiation patterns. *Journal of Rehabilitation Medicine.* 2006;38(5):287-294.

**25.** Lamontagne A, Malouin F, Richards CL. Locomotor-specific measure of spasticity of plantarflexor muscles after stroke. *Archives of Physical Medicine and Rehabilitation.* 2001;82(12):1696-1704.

**26.** Duncan PW, Samsa GP, Weinberger M, et al. Health status of individuals with mild stroke. *Stroke.* 1997;28(4):740-745.

**27.** Nelson RM, Currier DP. *Clinical electrotherapy.* Appleton & Lange; 1991.

**28.** O'Sullivan SB, Schmitz TJ. *Physical Rehabilitation* 5th ed. Philadelphia: F.A. Davis Company 2007.

**29.** Barth E, Herrman V, Levine P, Dunning K, Page SJ. Low-dose, EMG-triggered electrical stimulation for balance and gait in chronic stroke. *Topics in stroke rehabilitation.* 2008;15(5):451-455.

**30.** Yavuzer G, Geler-Kulcu D, Sonel-Tur B, Kutlay S, Ergin S, Stam HJ. Neuromuscular electric stimulation effect on lower-extremity motor recovery and gait kinematics of patients with stroke: a randomized controlled trial. *Archives of Physical Medicine and Rehabilitation.* 2006;87(4):536-540.

**31.** Ansari NN, Naghdi S, Arab TK, Jalaie S. The interrater and intrarater reliability of the Modified Ashworth Scale in the assessment of muscle spasticity: limb and muscle group effect. *NeuroRehabilitation.* 2008;23(3):231-237.

**32.** Matthews WB. Ratio of maximum H reflex to maximum M response as a measure of spasticity. *Journal of Neurology, Neurosurgery, and Psychiatry.* 1966;29(3):201-204.

**33.** Martins FL, Carvalho LC, Silva CC, Brasileiro JS, Souza TO, Lindquist AR. Immediate effects of TENS and cryotherapy in the reflex excitability and voluntary activity in hemiparetic subjects: a randomized crossover trial. *Revista Brasileira De Fisioterapia.* 2012;16(4):337-344.

**34.** Winter DA, Scott SH. Technique for interpretation of electromyography for concentric and eccentric contractions in gait. *Journal of electromyography and kinesiology : official journal of the International Society of Electrophysiological Kinesiology.* 1991;1(4):263-269.

**35.** Bilney B, Morris M, Webster K. Concurrent related validity of the GAITRite® walkway system for quantification of the spatial and temporal parameters of gait. *Gait & Posture.* 2003;17(1):68-74.

**36.** McDonough AL, Batavia M, Chen FC, Kwon S, Ziai J. The validity and reliability of the GAITRite system's measurements: A preliminary evaluation. *Archives of Physical Medicine and Rehabilitation.* 2001;82(3):419-425.

**37.** Menz HB, Latt MD, Tiedemann A, Mun San Kwan M, Lord SR. Reliability of the GAITRite walkway system for the quantification of temporo-spatial parameters of gait in young and older people. *Gait & Posture.* 2004;20(1):20-25.
