# Supplementary material for: Effects of neuromuscular electrical stimulation on gait performance in chronic stroke with inadequate ankle control - A randomized controlled trial
Source: PLoS One. 2018 Dec 10;13(12):e0208609. doi: 10.1371/journal.pone.0208609 (PMC6287810; doi:10.1371/journal.pone.0208609)
Supplement: S3 File — (DOCX) [file pone.0208609.s003.docx]

**Effects of Neuromuscular Electrical Stimulation on Gait Performance in Chronic Stroke with Inadequate Ankle Control - A Randomized Controlled Trial**

**(IRB title: The effects of neuromuscular electrical stimulation on ankle control and gait performance in individuals with chronic stroke：A randomized controlled trial)**

**Chapter 1 Introduction**

**Section 1 Background and motivation**

Gait dysfunctions resulting from spasticity, muscle weakness, and poor motor control are the leading causes of chronic physical disability and safety problems in individuals with stroke. Deceased gait velocity and asymmetry gait pattern are frequently reported after a stroke, and inadequate ankle control during walking has been shown to be one of the key reasons contributing to these gait dysfunctions. Muscle weakness of ankle dorsiflexors and spasticity of plantarflexors could result in insufficient ankle dorsiflexion at heel strike and in the swing phase during walking, and muscle weakness and spasticity of ankle plantarflexors could lead to insufficient ankle plantarflexion in terminal stance or push off. Traditional strategies include muscle performance training, flexibility exercise, hypertonia muscle management, locomotion training, and modalities. Neuromuscular electrical stimulation (NMES), which provides ES on nerve fibers in healthy or denervated muscles to induce muscle contractions, is the most widely used modality in clinics and has been adopted to manage the drop foot in stroke patients. Although the outcome of utilizing NMES in stoke rehabilitation is promising, studies that examined the effect of NMES on dynamic spasticity during walking and gait asymmetry in stroke patients were limited.

**Section 2 Purpose**

The purpose of this study was to investigate the effects of applying NMES over ankle dorsiflexors or plantarflexors on ankle control during walking and gait performance in chronic stroke patients.

**Section 3 Hypothesis**

We hypothesized that the effects of applying NMES over ankle dorsiflexors was more dominant than the effects of applying NMES over ankle plantarflexors.

**Section 4 Importance**

This information can provide the possible mechanisms of NMES involved in ankle control during walking and enhance the development of training strategy in stroke rehabilitation.

**Chapter 2 Literature review**

**Section 1 Gait dysfunctions in stoke**

Inadequate ankle control during walking has been identified as one key factor contributing to gait dysfunction, such as decreased gait speed and symmetry after stroke. Many stroke rehabilitation programs have thus targeted on the improvement of ankle control.

**Section 2 Effects of muscle weakness and spasticity of ankle plantarflexors on gait**

Weakness of ankle dorsiflexors with or without plantarflexors spasticity could result in insufficient ankle dorsiflexion at heel strike and in swing phase during walking, and muscle weakness and spasticity of ankle plantarflexors could lead to insufficient ankle plantarflexion in terminal stance or push off. Studies have shown that spasticity of ankle plantarflexors associated with the decreased gait velocity and increased gait asymmetry in stroke patients. Dynamic spasticity of ankle plantarflexors, which is locomotor-specific, was positively correlated with gait velocity and could explain 53% of the variance in spatial gait asymmetry.

**Section 3 Effects of NMES on stroke gait**

Neuromuscular electrical stimulation (NMES), which provides ES on nerve fibers in healthy or denervated muscles to induce muscle contractions, is the most widely used modality in clinics and has been adopted to manage the drop foot for stroke patients since early 1960s. The high peak current with specific waveform of NMES can maximize the numbers of responding motor units and their firing rate leading to tetantic contractions and great forces. Applying NMES may not only improve muscle strength but decrease spasticity of agonist or antagonist muscles possibly through maximal contraction inducing relaxation or reciprocal inhibition respectively.

**Chapter 3 Research method**

**Section 1 Design**

This will be a single-blinded, parallel randomized, controlled trial study. Participants will be randomized to one of three groups using block randomization with a block size of three: the NMES-TA group (NMES applied on tibialis anterior muscle), the NMES-MG group (NMES applied on medial gastrocnemius muscle), and the control group. Participants in the NMES groups will receive 20 minutes of NMES on either TA (NMES-TA) or MG (NMES-MG) and then 15 minutes of ambulation training. Participants in the control group will receive 20 minutes of range of motion and stretching exercises, followed by 15 minutes of ambulation training. Ambulation training will be focused on ankle movement and ankle control with verbal cues. All training sessions will occur 3 times per week for 7 weeks. The pre- and post-training assessments will be obtained by another physical therapist who was blinded to the group assignment.

**Section 2 Method**

**2.1 Participants**

To be included in the study, participants with stroke will have to satisfy the following criteria: (1) diagnosis of first-ever stroke with unilateral motor deficits at least 6 months, (2) with inadequate ankle control during gait (defined as dorsiflexion less than -5° at heel strike and plantarflexion less than 10° in push off in this study), (3) with passive range of motion (PROM) of ankle dorsiflexion at least to neutral position (defined as 0°), (4) ability to walk at least 10 m with or without assistive devices, and (5) a detectable surface EMG signal (>5 μV) from the tibialis anterior (TA) and medial gastrocnemius (MG) muscles of the affected leg. The exclusion criteria included (1) surface sensory loss of affected lower leg, (2) insufficient cognition to communicate (Mini-Mental State Examination < 24), (3) contraindications to NMES, such as a pacemaker or tumor, and (4) a history of orthopedic or other neurologic disorders affecting walking function.

**2.2 Protocol**

Participants who had suffered a stroke will be recruited from the community and medical centers. The diagnosis, age, sex, stroke type, lesion side, and post onset duration of stroke will be obtained from patient interviews and medical charts.

**2.3 Assessment**

**2.3.1 Spastisity**

**1. Manual testing**

Static spasticity of ankle plantarflexors will be measured by Modified Ashworth Scale in supine position.

**2. H reflex**

H reflex of the posterior tibial nerve will be evaluated by Medelec EMG/EP Diagnostic Instruments to identify the spasticity of ankle plantarflexors.

**3. Dynamic spasticity**

Dynamic spasticity of ankle plantarflexors in stance phase during gait will be evaluated using the method proposed by Lamontagne et al. in 2001.

**2.3.2 Gait analysis**

Spatial-temporal parameters of gait will be obtained from the GAITRite system.

**2.3.3. Muscle strength**

**1. Static muscle strength**

Isometric muscle strength of ankle dorsiflexor and plantarflexor of the affected limb will be measured by a handheld dynamometer.

**2. Dynamic muscle strength**

The electromyographic activity of tibialis anterior and gastrocnemius will be measured during gait by BIOPAC Data Acquisition System.

**2.3.4 Ability of ankle control**

Ankle range of motion will be measured by twin-axis electronic goniometer at heel strike and push off during gait.

**2.4 NMES training**

An EMG-triggered NMES (Myomed 932, Enraf Nonius, Netherlands) with two surface electrodes will be used for electrical stimulation. Participants in NMES groups will receive 20 minutes NMES on tibialis anterior or gastrocnemius followed by 15 minutes of ambulation training 3 times per week for 7 weeks. The frequency of NMES is set at 50 Hz with a 0.2 ms pulse width. Biphasic square wave is chosen, and the stimulation duty cycle is 5:15 (on:off) in seconds for 20 minutes. The intensity of stimulation is set from 50 mV to 0 mV. The NMES is triggered by electromyographic biofeedback devices. EMG signals of maximal voluntary contraction of ankle dorsiflexion subtracting 2 uV will be used as the initial training goal in every session. The participants will be asked to actively dorsiflex or plantarflex to reach the training goal to activate the electrical stimulation. After participants completed 5 cycles of active ankle dorsiflexion, the training goal will be increased by 2 uV progressively and lasted for 20 minutes.

**2.5 Statistical analysis**

A Wilcoxon signed-rank test was used to detect the pre- and post-training differences for within-group comparisons. To evaluate the training effects for between-group comparisons, a Kruskal-Wallis test followed by Dunn-Bonferroni post-hoc pairwise comparison was conducted for percentage of change score after training. Statistical significance is set at p < .05. All statistical analyses will be performed with the SPSS 20.0 software (SPSS Inc., USA).
